# Empathic Accuracy in Female Adolescents with Conduct Disorder and Sex Differences in the Relationship Between Conduct Disorder and Empathy

**DOI:** 10.1007/s10802-020-00659-y

**Published:** 2020-06-02

**Authors:** N. A. Martin-Key, G. Allison, G. Fairchild

**Affiliations:** 1 Cambridge Centre for Neuropsychiatric Research, Department of Chemical Engineering and Biotechnology, University of Cambridge, Cambridge, UK; 2 School of Psychology, University of Southampton, Southampton, UK; 3 Department of Psychology, University of Bath, Bath, UK

**Keywords:** Empathy, Emotion recognition, Empathic accuracy, Conduct disorder, Callous-unemotional traits, Sex differences

## Abstract

Research on empathy in youth with Conduct Disorder (CD) has primarily focused on males, with the few studies that have investigated empathy in females relying on questionnaire measures. Our primary aim was to investigate whether females with CD show empathy deficits when using a more ecologically-valid task. We used an empathic accuracy (EA) paradigm that involved watching videos of actors recalling emotional experiences and providing continuous ratings of emotional intensity (assessing EA), naming the emotion expressed (emotion recognition), and reporting whether they shared the emotion expressed (affective empathy). We compared 23 females with CD and 29 typically-developing (TD) adolescents aged 13–18 years. The CD sample was divided into subgroups with higher (CD/CU+) versus lower (CD/CU−) levels of callous-unemotional traits. Females with CD did not differ from TD females in EA or emotion recognition but exhibited reduced affective empathy responses (*p*s < 0.01, *r*s ≥ 0.39). The CD/CU+ and CD/CU− subgroups did not differ on any empathy measure. We also assessed for sex differences in CD-empathy associations by comparing the present data with archive data from males. CD adolescents exhibited impairments in EA relative to their TD counterparts overall (*p* < 0.05, ηp^2^ = 0.06), but there was no sex-by-diagnosis interaction. While females with CD were only impaired in affective empathy, males with CD exhibited emotion recognition and affective empathy deficits. This study demonstrates that females with CD show relatively specific impairments in affective empathy on an ecologically-valid task, whereas males with CD display more global empathic difficulties.

**Electronic supplementary material:**

The online version of this article (10.1007/s10802-020-00659-y) contains supplementary material, which is available to authorized users.

## Introduction

Empathy has been defined as the ability to share another’s affective state (Bernhardt and Singer 2012) and can be fractionated into at least two distinct forms: cognitive empathy or emotion recognition (i.e., recognizing and understanding others’ emotions) and affective empathy (i.e., sharing another person’s emotions; Blair 2005). Despite this distinction, it has been proposed that these two forms of empathy are strongly related, and that understanding another’s emotions is required in order to share their affective state (Gonzalez-Liencres et al. 2013). Empathy has been studied extensively in antisocial populations, with prior research finding that adolescents with Conduct Disorder (CD), a psychiatric condition characterized by aggression and antisocial behavior, exhibit emotion recognition and empathic deficits (e.g., Anastassiou-Hadjicharalambous and Warden 2008; Bowen et al. 2013; De Wied et al. 2012; Fairchild et al. 2009). A limitation of the literature on empathy in antisocial populations, and children and adolescents with CD or related constructs such as psychopathic or callous-unemotional (CU) traits, in particular, is that it has focused primarily on males. Furthermore, findings in this area have been inconsistent and many of these studies have employed questionnaire measures of dispositional empathy, which may be subject to demand characteristics, or highly simplified stimuli or tasks. In addition, many earlier studies have failed to account for the multi-dimensional nature of empathy and the possibility that different disorders may be linked to deficits in distinct aspects of empathy (e.g., intact cognitive empathy, but reduced affect sharing; Coll et al. 2017).

In a large questionnaire study with a community sample of healthy children, Dadds et al. (2009) found that psychopathic traits were negatively associated with both cognitive and affective empathy in boys, but only cognitive empathy in girls, suggesting that the effects of psychopathic traits are more global or pervasive in males relative to females. In a similar study with a longitudinal design, Brouns et al. (2013) found a significant negative association between psychopathic traits and cognitive empathy in females, but not in males. Interestingly, the authors also found that adolescents with moderate levels of psychopathic traits had lower affective empathy scores than those with low levels of such traits, irrespective of sex.

Critically, to our knowledge, studies that have used laboratory-based tasks to study empathy in antisocial populations have focused almost exclusively on males. For instance, in an all-male sample of adolescents with disruptive behaviour disorders (DBDs) who were classified as being either high (DBD/CU+) or low (DBD/CU−) in CU traits, De Wied et al. (2012) found that self-reported empathic responses to positive video clips were weaker in both DBD subgroups relative to controls. Furthermore, both DBD subgroups exhibited blunted facial electromyography responses to sad video clips, whereas the DBD/CU− group also showed a reduced facial electromyography response to happy video clips. On the other hand, the DBD/CU+ group showed weaker heart rate responses to sad clips relative to both the DBD/CU− and the control groups, although no group differences were seen for the other video clips.

Similarly, in a primarily male sample, Anastassiou-Hadjicharalambous and Warden (2008) found that children with CD and high levels of CU traits showed reduced heart rate responses to a single video-clip depicting a fear-inducing situation relative to those with low levels of CU traits and healthy controls. Interestingly, both CD subgroups reported lower affective empathy responses to the video-clip than controls and obtained lower scores on a questionnaire measure of affective empathy. Considered together, these studies suggest that empathic deficits may be observed in both high and low CU traits subgroups, depending on the outcome measures used.

Critically, these laboratory-based studies selected excerpts from television shows or movies, meaning that the emotions displayed in the clips were inevitably artificial, and further, that it was not possible to determine whether the actors (targets) were genuinely feeling the emotions they were portraying. To overcome these limitations, Martin-Key et al. (2017) created an empathic accuracy (EA) paradigm, where EA is defined as the ability to accurately infer the thoughts and feeling of another person, including changes in the intensity of their emotions on a moment-to-moment basis (Ickes et al. 1990; Zaki et al. 2009). A key feature of the paradigm is that targets were filmed talking about emotionally-charged autobiographical experiences, and subsequently rated the intensity of the emotions they experienced on a continuous basis during the clip. The participants in the study were asked to watch the clips in question (which were differentiated into happy, sad, angry, fearful, disgusted, or surprised experiences) and provided continuous ratings of the target’s emotional intensity (the concordance between the target’s and the perceiver’s ratings is the key measure of EA). They also identified the emotion that the target was experiencing (emotion recognition) and reported the emotion that they experienced themselves while watching the film (affective empathy). Relative to healthy controls, the CD group showed poorer recognition and reduced affective empathy for sadness, fear, and disgust, but there were no significant differences in EA. Importantly, this study, along with Brook and Kosson’s (2013) study of EA in adult offenders, was restricted to males alone.

Collectively, these studies suggest that CD or DBDs in general are associated with deficits in empathy, but experimental studies on empathy in such populations are rare, and to our knowledge, no prior studies have investigated empathy in females with CD or DBDs using laboratory-based tasks, rather than questionnaires. The only studies that explicitly investigated sex differences in empathy (Brouns et al. 2013; Dadds et al. 2009) used questionnaire measures assessing cognitive and affective empathy and focused on associations with psychopathic traits, rather than CD or ODD as a diagnosis. It is therefore important to extend the evidence base by examining whether females with CD show empathy deficits when more objective and ecologically-valid measures are used, as this could have implications for treatment and the development of emotion and empathy training programs. Another key question is whether the effects of variation in CU traits are similar or different in females compared to males with CD, given prior evidence that psychopathic traits may show distinct associations with affective or cognitive empathy in typically-developing males compared to females.

To address these questions, we assessed EA, emotion recognition, and affective empathy in females with CD using the same paradigm employed by Martin-Key et al. (2017). To ensure comparability with the earlier study and because females typically develop CD in adolescence, as opposed to childhood (Silverthorn and Frick 1999), we recruited females aged between 13 and 18 years. In line with the findings of Martin-Key et al. (2017), we predicted that group differences would be most evident for emotion recognition and affective empathy, with negative emotions such as sadness, fear or disgust likely to be particularly affected in females with CD. Although Martin-Key et al. (2017) did not find significant group differences in EA in their study on males, we tested for EA deficits in females with CD, predicting that, *if present*, these would be most evident for negative emotions.

In addition, we examined whether CU traits influenced EA task performance within the CD group, predicting that, *if subgroup differences were present*, females with CD and higher levels of CU traits (CD/CU+) would show reduced EA, emotion recognition, and affective empathy relative to CD females with lower levels of CU traits (CD/CU+). We hypothesized that empathy for sadness and fear would be disproportionately affected, given that CU traits have been linked to impaired recognition of distress cues (e.g., Blair and Coles 2000; Fairchild et al. 2010).

Our third objective was to test for sex differences in EA task performance. To do this, we used archive data obtained from male CD and TD participants. On the basis of prior research on facial emotion recognition, we predicted that males and females with CD would show similar deficits in emotion recognition and affective empathy. As only a very limited number of studies have investigated EA in antisocial populations (Brook and Kosson 2013; Martin-Key et al. 2017), and all of these studies recruited male-only samples, it was not possible to make clear predictions about sex differences in EA.

## Method

### Participants

Fifty-two female adolescents (23 CD, 29 TD) aged 13–18 years were recruited through Youth Offending Services and pupil referral units via referrals from caseworkers, and through mainstream schools and colleges via mail-shots in the Hampshire region of the UK. Exclusion criteria included the following: Intelligence Quotient (IQ) <70, as estimated using the Wechsler Abbreviated Scale of Intelligence (WASI; Wechsler 1999), the presence of ASDs, psychosis, and severe current bipolar or mood disorder. All participants and the parents of those aged below 16 provided written informed consent. Those aged below 16 were also asked to indicate their assent. This study was approved by the University Ethics Committee and the Hampshire County Council Research and Evaluation Unit.

### Measures

#### The Schedule of Affective Disorders and Schizophrenia for School-Aged Children (K-SADS)

All participants were assessed for CD, Oppositional Defiant Disorder (ODD), Attention-Deficit/Hyperactivity Disorder (ADHD), Major Depressive Disorder (MDD), Generalised Anxiety Disorder (GAD), Obsessive-Compulsive Disorder, Post-Traumatic Stress Disorder (PTSD), Psychosis, and Alcohol and Substance Use Disorders using the K-SADS (Kaufman et al. 1997). Autistic spectrum disorders were assessed using the ASD module of the DSM-5 version of the K-SADS. Diagnostic interviews were carried out separately with participants and caregivers, and a symptom was considered present if it was endorsed by either informant, following Kaufman et al. (1997). The inter-rater reliability of diagnoses of CD and other common disorders ranged from Cohen’s kappa = 0.87 to 1.00 (*n* = 50), indicating excellent agreement between raters.

#### The Inventory of Callous-Unemotional Traits

CU traits were assessed using the self-report Inventory of Callous-Unemotional traits (ICU; Frick 2003; Cronbach’s alpha in the present sample = 0.78). The ICU is a 24-item measure requiring participants to indicate whether they agree with statements such as ‘I do not care who I hurt to get what I want’ on a four-point Likert scale, ranging from 0 = ‘not at all true’ to 3 = ‘definitely true’. Higher scores reflect higher CU traits.

Within the CD group, participants were classified as being higher (CD/CU+) or lower (CD/CU−) in CU traits using a median split procedure based on total ICU scores. Participants scoring >28 were classified as CD/CU+, whereas those scoring ≤28 were classified as CD/CU−. This approach was selected to increase the comparability of our work with earlier research (e.g., De Wied et al. 2012; Jones et al. 2010; Martin-Key et al. 2017; Schwenck et al. 2012). Nevertheless, as this approach has several limitations (e.g., loss of power; MacCallum et al. 2002), we also tested for correlations between CU traits and EA, cognitive empathy, and affective empathy within the CD and TD groups separately, as well as across the entire sample.

#### The Interpersonal Reactivity Index

To provide continuity with the previous literature on empathy in adolescents with CD, we also included a measure of dispositional empathy: the self-report Interpersonal Reactivity Index (IRI; Davis 1983; Cronbach’s alpha in the present sample = 0.66). Participants were required to rate their agreement with statements such as ‘Sometimes I don’t feel very sorry for other people when they are having problems’ on a 5-point Likert scale ranging from 0 = ‘does not describe me very well’ to 4 = ‘describes me very well’. This questionnaire includes four subscales (each containing seven items) assessing different aspects of empathy, i.e., perspective taking, fantasy, empathic concern, and personal distress. Higher scores indicate higher levels of dispositional empathy.

#### Demographic Characteristics

The ethnicity of the participants was categorized as either Caucasian or non-Caucasian and socioeconomic status (SES) was classified as either high or low on the basis of parental occupation using the UK Office of National Statistics (ONS 2010) guidelines.

#### Empathic Accuracy Task

This task evaluated whether participants could: (a) consistently track changes in the intensity of the target’s emotion (EA); (b) identify the target’s emotion (emotion recognition/cognitive empathy); and (c) experience the same emotion as the target (affective empathy). The task was modified from a paradigm developed by Zaki et al. (2009). The creation of the stimulus material and alterations to the task design are described in detail in the Online Supplementary Materials. In brief, actors (targets) were asked to recall autobiographical events in which they had experienced one of the six basic emotions (anger, happiness, sadness, disgust, fear, and surprise) strongly and where it had been a relatively ‘pure’ emotion (i.e., not accompanied by other strong emotions). After writing a brief description of the event, the actors then discussed the experience with the researcher, with emphasis on re-experiencing the emotion they had felt at the time, at which point they were filmed talking aout the event (without naming the target emotion, e.g., ‘I felt sad’). Immediately after filming, the actors watched the clip and provided continuous ratings of the strength of their emotions from 0 = *no emotion* to 9 = *very strong emotion*. It was emphasized that they should rate how they felt *while speaking about the event*, rather than during the event itself.

### Procedure

**Fig. 1:**
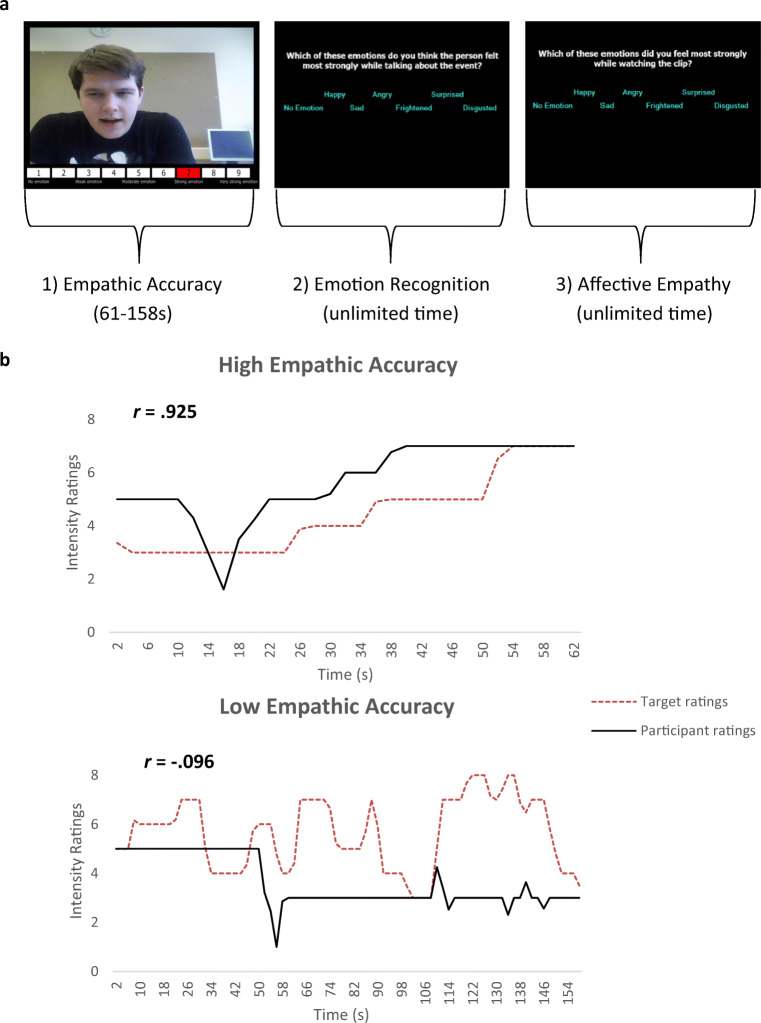
Schematic representation of a trial sequence of the empathic accuracy task (panel **a**) and example correlations between the perceiver’s and the target’s continuous ratings of emotional intensity (panel **b**)

The participants were required to watch two practice clips to familiarize themselves with the task and rating scale, and then watched 12 test clips comprising two instances of each of the following emotions: anger, happiness, sadness, disgust, fear, and surprise. These clips lasted between 61 and 158 s with a mean length of 144 s. During the presentation of each video clip, participants were asked to rate, on a continuous basis, the intensity of the emotions being experienced by the target using the same rating scale as described above (Fig. 1a). We computed the correlations between the targets’ continuous ratings of the intensity of their emotions and the perceivers’ ratings of emotional intensity on the same scale. The correlations between the targets’ and perceivers’ continuous ratings formed the dependent measure of EA (see Fig. 1b for examples of low and high correlations). After each clip, participants were asked to identify the emotion displayed in the video-clip from a list of the six primary emotions. There was also an option of ‘*no emotion*’. Participants also named the predominant emotion *they* experienced whilst watching the clip (again, with options of the six primary emotions and ‘*no emotion*’) – indexing affective empathy.

### Data Analyses

#### CD Vs. TD Females

Continuous EA data for each participant and clip were downsampled. Mean ratings for each two-second period served as one data point (bin) in subsequent analyses. Participants’ ratings of emotional intensity across all bins were correlated with the target’s own ratings. Average correlations for each participant per emotion were then calculated. Correlations were compared between groups using a 2 (Group: CD vs. control; CD/CU+ vs. CD/CU−) × 6 (Emotion: sadness, happiness, fear, surprise, anger, disgust) ANOVA.

For emotion recognition, performance accuracy was considered for each emotion separately as the data were non-normally distributed and could not be transformed to a normal distribution. Participants could receive scores of 0, 50, or 100% for each emotion (correct emotion identified in 0/2, 1/2, or 2/2 clips, respectively). Emotion recognition scores for each emotion were compared between groups (CD vs. control; CD/CU+ vs. CD/CU−) using Mann-Whitney *U* tests, applying the Holm-Bonferroni correction for multiple comparisons (Holm 1979).

Similar procedures were used to measure affective empathy as these data were not normally distributed; participants could obtain scores of 0, 50, or 100% for affect matches for each emotion (the target’s emotion shared in 0/2, 1/2, or 2/2 clips, respectively). Affective empathy scores for specific emotions were compared between groups (CD vs. control; CD/CU+ vs. CD/CU−) using Mann-Whitney *U* tests, again applying the Holm-Bonferroni correction. Effect sizes are reported either as *r*_equivalent_ for the direct group comparisons (Rosenthal and Rubin 2003; small ≥ 0.10, medium ≥ 0.30, large ≥ 0.50; Cohen 1988) or partial eta squared (η_p_^2^) for the ANOVAs (small ≥ 0.01, medium ≥ 0.06, large ≥ 0.14; Cohen 1988).

#### Sex Differences in EA Task Performance

Archive data from our previous study on males (Martin-Key et al. 2017) were used to assess whether the effects of CD on EA, emotion recognition, and affective empathy are different in males and females. We selected the 52 male participants who best matched the female sample in terms of age, IQ, SES, and psychiatric comorbidity (see Supplementary Table S4 for sample characteristics).

Main effects of diagnosis and group on EA, as well as sex-by-diagnosis interactions, were tested by running a 2 (Group: CD vs. control) × 2 (Sex: male vs. female) × 6 (Emotion: sadness, happiness, fear, surprise, anger, disgust) mixed-design ANOVA. Given that the emotion recognition and affective empathy data were not normally distributed, Kruskal-Wallis *H* tests were used to investigate differences between the four groups (TD males, CD males, TD females, and CD females) on these measures. Post-hoc Mann-Whitney *U* tests, subject to Holm-Bonferroni correction, were conducted to follow up significant Kruskal-Wallis tests.

## Results

### Demographic and Clinical Characteristics: CD Vs. TD Group Comparisons in Females

Demographic characteristics and rates of psychiatric comorbidity by group are presented in Table 1, along with group comparisons. The female CD and control groups did not differ significantly in age, IQ, SES, or ethnicity. As expected, females with CD had significantly higher levels of CU traits than controls, *t* (49) = 3.96, *p* < 0.001, *r*_equivalent_ = 0.49. Eight CD participants had comorbid psychiatric disorders. However, 65% of the CD group were free of current comorbid disorders. No differences were found between the TD and CD groups in rates of mood disorders, *p* > 0.20; Fisher’s Exact Test (FET), but CD females had significantly higher rates of anxiety disorders, *p* < 0.05; FET. As none of the controls had ADHD, we could not test for differences between the groups in rates of ADHD diagnoses.

**Table 1:** Demographic characteristics and comorbidity: female CD vs. TD comparisons

	TD (*n* = 29)	CD (*n* = 23)	
	*M* (SD)		*t*
Age (years)	16.22 (1.94)	16.06 (1.63)	0.30
Estimated IQ	100.17 (12.66)	93.52 (16.11)	1.67
Callous-unemotional traits (ICU)	19.19 (6.95)	28.00 (8.59)	−3.96^***^
Empathy questionnaire (IRI)			
Perspective taking	15.79 (5.04)	12.55 (3.98)	2.41^*^
Fantasy	14.72 (5.07)	11.45 (5.56)	2.14^*^
Empathic concern	17.48 (3.61)	16.30 (2.90)	1.22
Personal distress	11.24 (2.92)	11.55 (4.43)	−0.29
Total IRI	65.10 (8.69)	56.60 (12.28)	2.84^**^
	*n* (%)		*χ*^*2*^
Socioeconomic status^≠^			
Higher	15 (52)	8 (35)	1.39
Lower	10 (34)	11 (48)	
Missing	4 (14)	4 (17)	
Ethnicity			
Caucasian	26 (90)	22 (96)	0.65
Non-white	3 (10)	1 (4)	
Psychiatric comorbidity			
ADHD	0 (0)	3 (13)	–
Mood disorder	1 (3)	3 (13)	1.66
Anxiety disorder	1 (3)	5 (22)	4.20^*^

^≠^Estimated on the basis of parental occupation using National Office of Statistics guidelines

^*^p < 0.05

^**^p < 0.01

^***^p < 0.001

Key: *ADHD* attention-deficit/hyperactivity disorder, *CD* Conduct Disorder, *ICU* Inventory of Callous-Unemotional traits, *IQ* intelligence quotient, *IRI* Interpersonal Reactivity Index, *TD* typically-developing

There were no group differences on the Empathic Concern and Personal Distress subscales of the IRI, but the CD group scored significantly lower on the Perspective Taking, *t* (47) = 2.41, *p* < 0.05, *r*_equivalent_ = 0.33, and Fantasy subscales, *t* (47) = 2.14, *p* < 0.05, *r*_equivalent_ = 0.30, and had lower total IRI scores, *t* (47) = 2.84, *p* < 0.01, *r*_equivalent_ = 0.38.

There were no differences between the CD/CU+ and CD/CU− subgroups in age, ethnicity, SES, IQ, or rates of ADHD and anxiety disorders, but the CD/CU+ participants had lower Empathic Concern subscale scores (*p* < 0.01, *r*_equivalent_ = 0.70; see Supplementary Table S2).

### Correlations Between Dispositional Empathy (IRI) and Empathic Accuracy, Emotion Recognition, and Affective Empathy

To further validate the EA task, we tested for associations between the key measures (EA, emotion recognition, and affective empathy) and total IRI scores, as well as the IRI subscales, in the total female sample. Empathic Concern scores and overall EA performance were positively correlated, *r* = 0.33, *p* < 0.05, as were Fantasy scores and overall affective empathy, *r* = 0.32, *p* < 0.05. However, IRI scores were not correlated with emotion recognition performance.

### EA Task Performance: CD Vs. TD Group Comparisons in Females

First, we tested for group differences in EA – i.e., participants’ ability to consistently track changes in emotional intensity displayed by the targets (see Table 2). Due to technical error, EA data from two subjects were lost, leaving 22 CD and 28 TD participants’ data available for analysis. The CD and TD groups did not differ significantly in EA (*F* (1, 48) = 2.03, *p* = 0.16, η_p_^2^ = 0.04) and there was also no Group-by-Emotion interaction (*F* (4.02, 192.85) = 0.89, *p* = 0.47, η_p_^2^ = 0.02).

**Table 2:** Empathic accuracy descriptive statistics: female CD vs. TD comparisons

Emotion	TD (*n* = 28^a^)	CD (*n* = 22^a^)
	Mean correlation (*r*) (SE)	Mean correlation (*r*) (SE)
Sadness	0.36 (0.04)	0.27 (0.07)
Happiness	0.42 (0.06)	0.33 (0.07)
Fear	0.34 (0.07)	0.35 (0.08)
Surprise	0.38 (0.06)	0.36 (0.08)
Anger	0.29 (0.05)	0.18 (0.07)
Disgust	0.33 (0.09)	0.10 (0.11)

^a^Empathic accuracy data were unavailable for one TD and one CD subject due to technical difficulties. Key: *CD* Conduct Disorder, *SE* standard error, *TD* typically-developing

Note*:* Mean scores were transformed back to correlation coefficient scores (*r*) from Fisher’s Z for ease of interpretation. Scores could range from −1 to 1 with higher scores representing higher levels of empathic accuracy

**Fig. 2:**
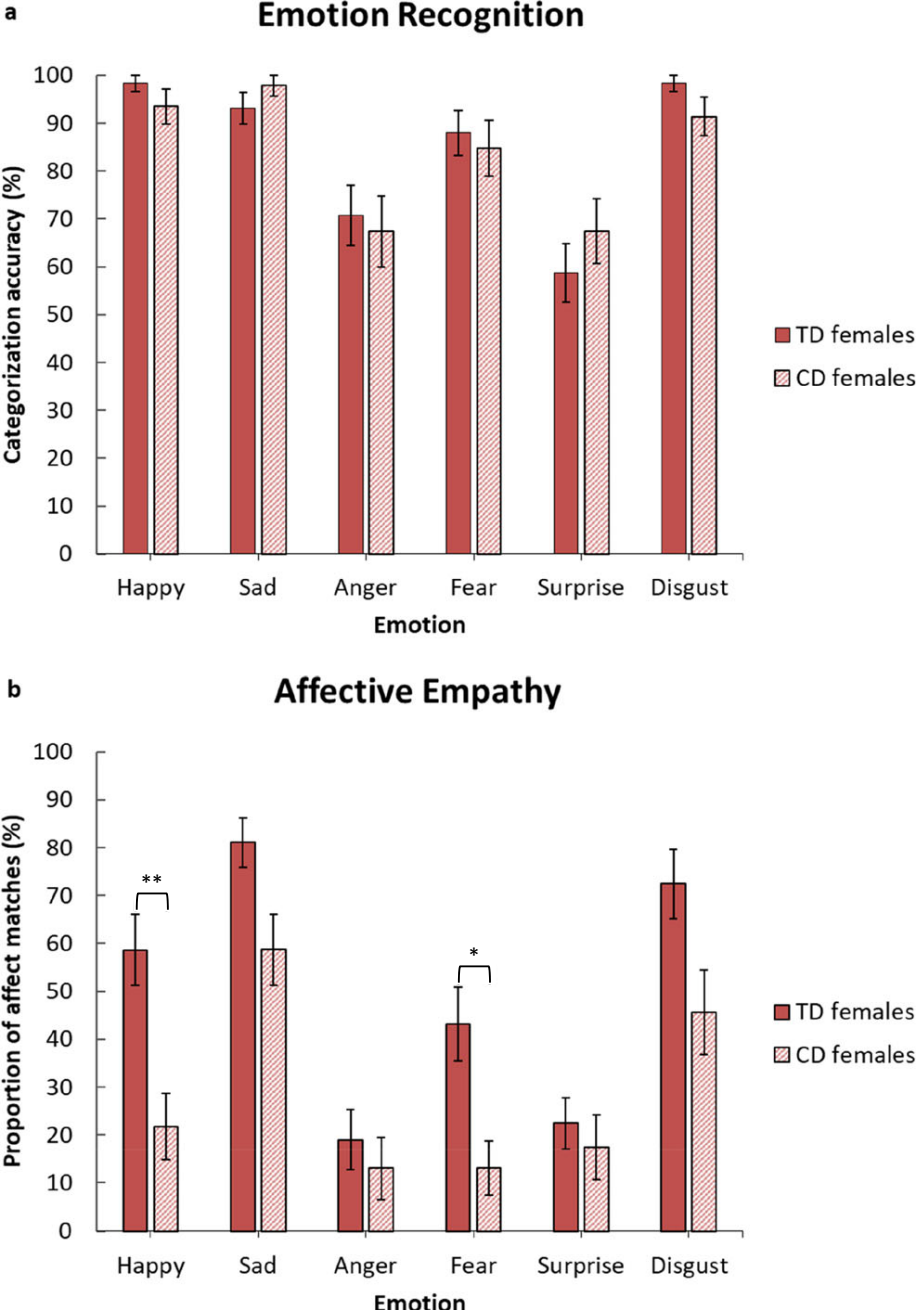
Mean emotion recognition scores (panel **a**) and mean affect matches to emotions displayed by targets (panel **b**) in the female typically-developing (*TD*) and Conduct Disorder (*CD*) groups (error bars show +/−Standard Error). The *p-*values shown are those obtained after applying the Holm-Bonferroni correction for multiple comparisons*;* *p < 0.05. **p < 0.01

In terms of emotion recognition, there were no significant differences between the CD and TD groups for any of the six emotions, all *ps* > 0.54; see Fig. 2a. However, relative to TD controls, participants with CD reported significantly fewer affect matches when watching clips depicting happiness, *U* = 170.50, *z* = −3.21, *p <* 0.01, *r*_equivalent_ = 0.45, and fear, *U* = 199, *z* = −2.79, *p* < 0.01, *r*_equivalent_ = 0.39, while there were trends towards the CD group reporting fewer affect matches for sadness, *p* = 0.07, *r*_equivalent_ = 0.33, and disgust, *p* = 0.07, *r*_equivalent_ = 0.32; see Fig. 2b. All of these group differences and trends had medium to large effect sizes.

To examine whether the affective empathy differences between the TD and CD groups were explained by comorbid disorders (e.g., ADHD or anxiety disorders) in the latter, we ran multiple regression analyses to test whether CD or other disorders were more important in explaining the observed group effects. Neither ADHD nor anxiety disorders were significant predictors of affective empathy for happiness or fear, all standardized *β*s < 0.16, *p*s > 0.23, suggesting that the main findings were not driven by group differences in psychiatric comorbidity. Having a diagnosis of CD was uniquely associated with reduced affective empathy for these emotions, standardized *β*s > −0.36, *p*s < 0.05, with CD accounting for ≥18% of the variance in affective empathy, all *R*^*2*^s > 0.18, *F*s > 2.61, *p*s < 0.05.

### EA Task Performance: CD/CU+ Vs. CD/CU− Group Comparisons in Females and Correlations Between CU Traits and EA Task Performance in TD Females

**Fig. 3:**
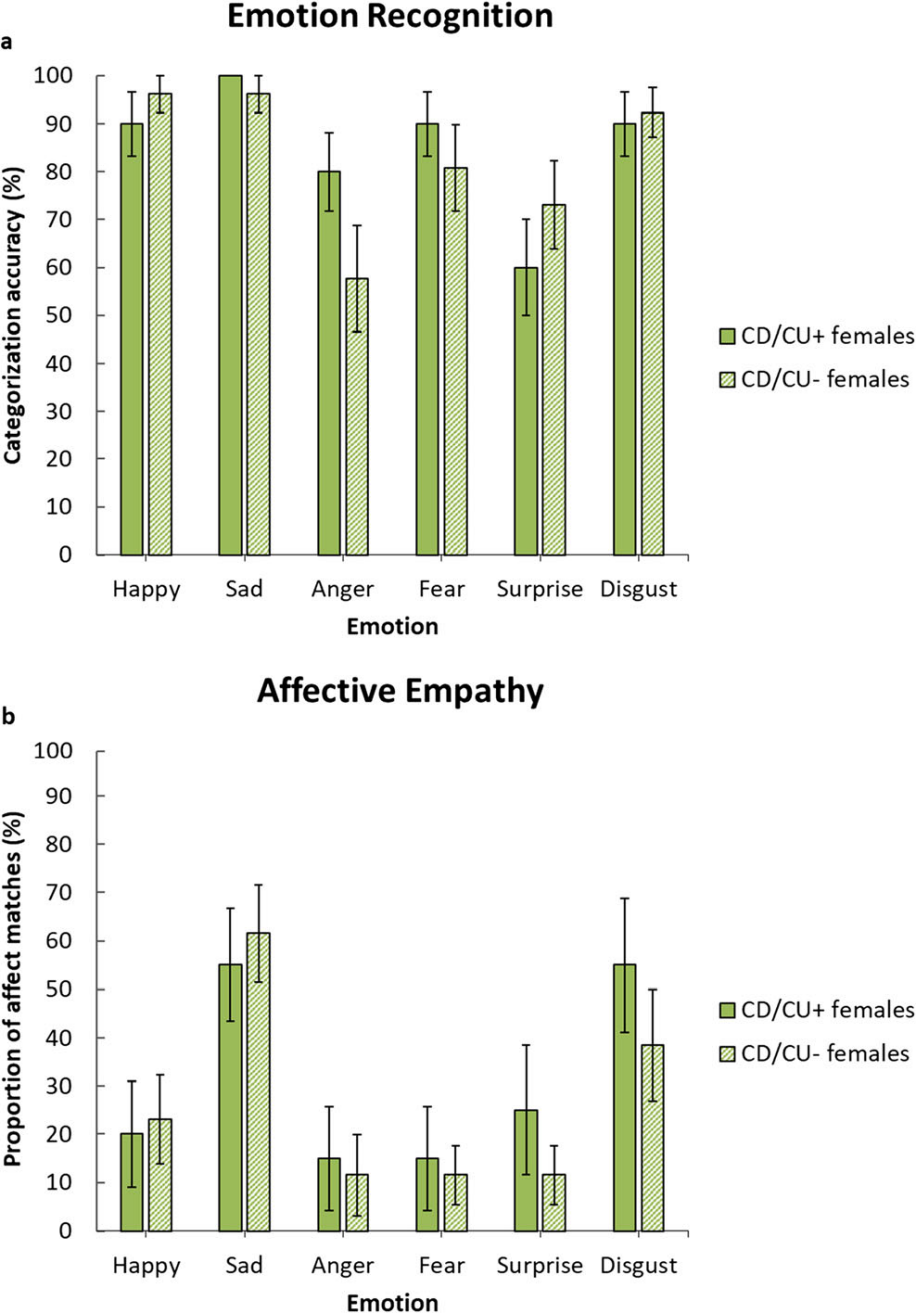
Mean emotion recognition scores (panel **a**) and mean affect matches to emotions displayed by targets (panel **b**) in the female Conduct Disorder subgroups with higher (CD/CU+) versus lower levels of callous-unemotional traits (CD/CU−)

The CD/CU+ and CD/CU− subgroups did not differ in EA (*F* (1, 20) = 1.01, *p* = 0.33, η_p_^2^ = 0.05) and there was no Group-by-Emotion interaction (*F* (5, 100) = 0.58, *p* = 0.72, η_p_^2^ = 0.03); see Supplementary Table S3). Furthermore, there were no significant differences between the subgroups in emotion recognition or affective empathy (all *p*s > 0.23; see Fig. 3).

**Fig. 4:**
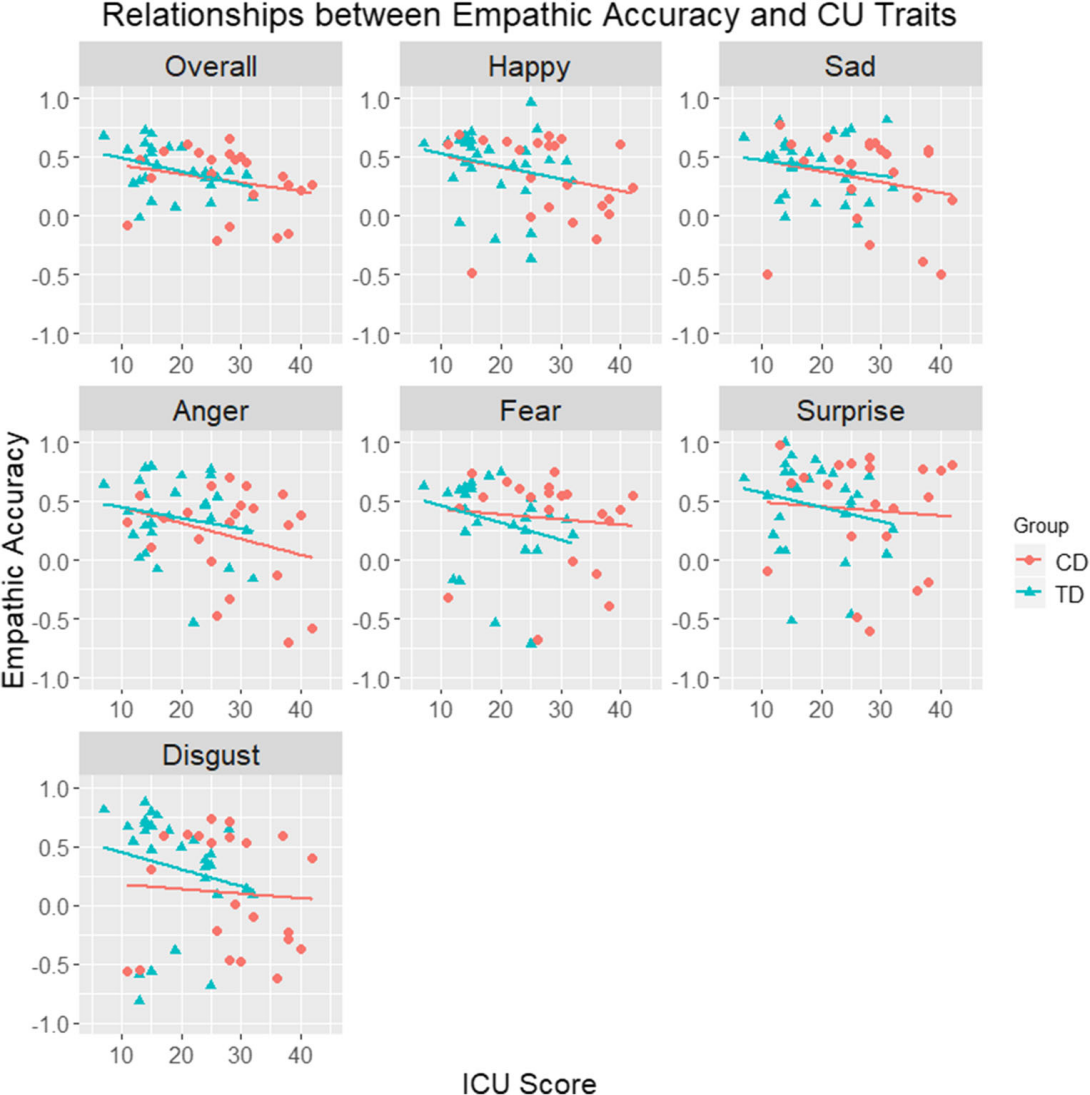
Relationships between empathic accuracy and CU traits across all emotions (overall) and per individual emotion, with separate regression lines for each group (CD vs. TD). Key: *CD* Conduct Disorder, *ICU* Inventory of Callous-Unemotional traits, *TD* typically-developing

When treating CU traits as a dimensional measure, no significant correlations between CU traits and EA, emotion recognition, or affective empathy were observed in the CD group (*ps* > 0.19). On the other hand, CU traits were negatively associated with affective empathy for happiness and sadness in the TD group (*rs* ≥ −0.41, *ps* < 0.05). Lastly, when considering the entire sample (collapsing across the CD and TD groups), CU traits were negatively associated with affective empathy for happiness and sadness (*r*s > −0.26, *ps* < 0.05), as well as lower EA scores across all emotions and for happy and angry clips specifically (*rs* > −0.32, *ps* < 0.05). See Fig. 4 for a visual representation of the associations between CU traits and EA.

### Sex Differences in EA Task Performance

When combining the data from females with archive data from males, we found a main effect of diagnosis on EA (*F* (1, 82) = 5.15, *p* < 0.05, η_p_^2^ = 0.06; see Supplementary Table S5), which was driven by poorer overall performance in the CD group. There was no main effect of sex or a sex-by-diagnosis interaction, suggesting a general CD-related deficit in EA.

**Fig. 5:**
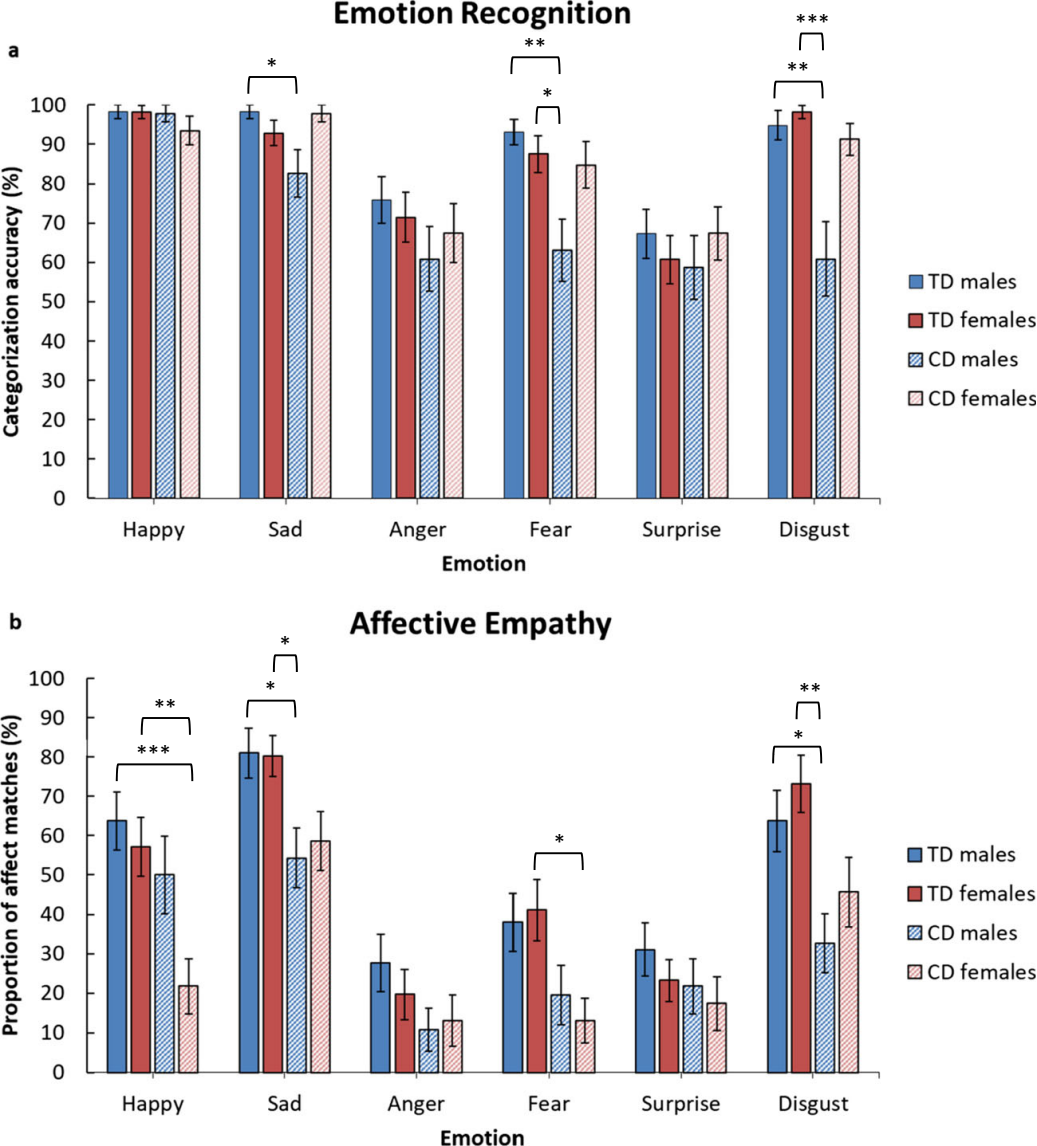
Mean emotion recognition scores (panel **a**) and mean affect matches to emotions displayed by targets (panel **b**) in the male typically-developing (*TD*), female TD, male Conduct Disorder (*CD*), and female CD groups (error bars show +/−Standard Error). The *p-*values shown are those obtained after applying the Holm-Bonferroni correction for multiple comparisons*;* *p < 0.05. **p < 0.01. ***p < 0.001

We then compared the four groups (TD males, CD males, TD females, CD females) in emotion recognition (see Fig. 5a). There were significant group differences for sadness (*H* (3) = 10.48, *p* < 0.05, *r*_equivalent_ = 0.72), fear (*H* (3) = 14.10, *p* < 0.01, *r*_equivalent_ = 0.81), and disgust recognition (*H* (3) = 22.38, *p* < 0.001, *r*_equivalent_ = 0.91). Mann-Whitney *U* tests revealed that CD males were worse than TD males at recognising sadness, fear, and disgust (*ps* < 0.05, *r*_equivalents_ ≥ 0.35). Similarly, in comparison to TD females, CD males were poorer at identifying fear and disgust (*ps* < 0.05, *r*_equivalents_ ≥ 0.36). However, there were no significant differences in emotion recognition between CD and TD females, or either of the female groups compared with TD males.

Finally, there were group differences in affective empathy for happiness (*H* (3) = 14.12, *p* < 0.01, *r*_equivalent_ = 0.81), sadness (*H* (3) = 13.64, *p* < 0.01, *r*_equivalent_ = 0.80), fear (*H* (3) = 11.65, *p* < 0.01, *r*_equivalent_ = 0.76), and disgust (*H* (3) = 14.09, *p* < 0.01, *r*_equivalent_ = 0.80; see Fig. 5b). Mann-Whitney *U* tests revealed that CD males reported significantly fewer affect matches for sadness and disgust than TD males and females (*ps* < 0.05, *r*_equivalents_ ≥ 0.35). Apart from the aforementioned differences in affective empathy between CD and TD females, the former group also reported fewer affect matches for happiness than TD males (*p* < 0.001, *r*_equivalent_ = 0.45). TD males and females did not differ in affective empathy.

## Discussion

The primary aim of the present study was to assess empathic accuracy (EA), emotion recognition, and affective empathy in female adolescents with CD and higher versus lower levels of CU traits, using a more ecologically-valid task than has been used previously. We also examined whether females and males with CD show similar or distinct impairments in EA, emotion recognition, and affective empathy. Relative to typically-developing (TD) females, females with CD showed reduced affective empathy when viewing emotionally-evocative video clips depicting real autobiographical experiences, particularly for fearful and happy experiences. Contrary to expectations, however, females with CD did not show emotion recognition deficits – even for negative emotions. Further, in line with the earlier study on males (Martin-Key et al. 2017), females with CD were not significantly impaired in their ability to consistently track changes in emotional intensity – although sample size and statistical power issues must be considered when interpreting this null finding.

The present findings for emotion recognition contradict our hypothesis, as well as earlier studies showing impaired emotion recognition in females with CD (e.g., Fairchild et al. 2010; Kohls et al. 2020). While issues with sensitivity and task complexity (i.e., ceiling effects) should be borne in mind when interpreting these findings, our findings should perhaps not be considered surprising given that the stimuli used in the present task contain visual, auditory, and linguistic information, and all were >60 s in duration. In line with this notion, it is important to note that not all studies have shown emotion recognition deficits in female with CD (Pajer et al. 2010), while another study found only weak evidence for emotion recognition impairments in females with conduct problems (CPs; Schwenck et al. 2014). Further, a recent study using dynamic and morphed static stimuli observed greater facial emotion recognition deficits in males with CD relative to their female counterparts (Martin-Key et al. 2018).

Critically, females with CD exhibited reduced affective empathic responses to the emotions displayed by others – such difficulties were particularly marked for fear and happiness, but were also present at a trend level for sadness and disgust. These findings are interesting, given that females with CD did not exhibit deficits in fear or happiness *emotion recognition*; the impairments were specific to *affective empathy*. In line with the earlier findings in males (Martin-Key et al. 2017), the overall pattern of affective sharing of different emotions was similar in the TD and CD groups, but simply shifted downwards in the latter.

To address our second aim, we investigated the impact of CU traits on empathy in females with CD by comparing the CD/CU+ and CD/CU− subgroups. Our findings revealed no differences between these subgroups for any of the EA task measures. Further, when treated as a dimensional measure, CU traits within the CD group were not significantly associated with any of the empathy measures. Interestingly, higher levels of CU traits across the entire sample were associated with reduced affect sharing when viewing happy and sad clips, although CU traits appeared to be more important in explaining variance within the TD group than the CD group. These findings suggest that CU traits may have a larger impact on affective empathy in individuals without diagnosable levels of CPs (i.e., CD). Furthermore, while we may not have had enough power to detect significant associations between CU traits and EA in the CD and TD groups separately, CU traits across the entire sample were associated with poorer EA performance across all emotions (overall EA) and for happy and angry clips specifically. Further studies with larger sample sizes are required in order to better understand the relationship between EA, conduct problems, and CU traits.

We also investigated whether females and males with CD show similar or distinct impairments in EA, emotion recognition, and affective empathy by analysing archive data from male CD and TD participants. These analyses revealed EA deficits in the combined CD group compared with the TD group; the fact that this was only evident in the *combined* analysis suggests that we had insufficient power to detect group differences in EA when conducting analyses in females and males separately. Critically, this novel finding builds upon the existing literature (e.g., Anastassiou-Hadjicharalambous and Warden 2008; Bowen et al. 2013; De Wied et al. 2012; Fairchild et al. 2009) by demonstrating that individuals with CD are not only likely to exhibit difficulties recognizing and sharing others’ emotions, but may also find it challenging to detect subtle changes in emotional intensity on a moment-to-moment basis (an important skill in real-life social situations).

Finally, in terms of emotion recognition and affective empathy, while females with CD only exhibited difficulties in affective empathy relative to healthy controls, males with the disorder demonstrated poorer emotion recognition *and* reduced affective empathy for negative emotions compared to TD controls. This suggests that males with CD are more likely to show global impairments in empathy than their female counterparts. These findings are in line with those obtained by Martin-Key et al. (2018), where having CD *and* being male resulted in additive, detrimental effects on emotion recognition performance for static and dynamic facial expressions. Taken together, the results of these studies highlight the fact that optimal intervention strategies aiming to remediate empathic deficits in this population are likely to differ by sex: a ‘lighter touch’ intervention may be sufficient for females with CD, as they are likely to present with less severe difficulties to start with.

### Strengths and Limitations

A key strength of this study was the use of a more ecologically-valid paradigm to assess distinct aspects of empathy and the use of video-clips portraying *discrete primary emotions*, rather than just positively- or negatively-valenced stimuli as in previous EA paradigms (Lee et al. 2011). The use of relatively naturalistic stimuli which include visual, auditory, and linguistic information means that our results should be more applicable to real-life social situations than those obtained using artificial or static stimuli. The assessment of EA, which indexes the individual’s ability to track dynamic changes in the intensity of another’s emotions, is another useful addition to the literature on empathy in CD and CU traits. Furthermore, a recent position paper argued that it is crucial to differentiate between emotion identification and affect sharing, as we have done in the present study, as these empathic processes are related, but may break down in different ways in different disorders (Coll et al. 2017). Finally, the present study is the first, to our knowledge, to directly compare males and females with CD using a lab-based measure of empathy.

Nonetheless, this study also had some limitations. Given the difficulty of recruiting females with CD, our sample size was relatively small (*N* = 52), meaning that some of the present findings could reflect false positives. On the other hand, the correction for multiple comparisons we applied may have been too conservative, leading to false negatives. Therefore, the findings from the current study should be interpreted with caution and require replication in larger samples. Another limitation relates to our measure of EA as it could be argued that using a single correlation to depict an individual’s ability to detect changes in emotional intensity may be overly simplistic and may not fully capture convergence rates between the target’s and the participant’s ratings on a moment-to-moment basis. Nevertheless, this approach of deriving a single correlation for each clip has been used in prior basic and clinical research on EA (Lee et al. 2011; Zaki et al. 2009), as well as the earlier study on EA in male adolescents with CD (Martin-Key et al. 2017). While we were specifically interested in investigating participants’ ability to *consistently* track changes in emotional intensity, future studies may need to explore alternative ways of conceptualising and measuring EA. These studies could also try to disentangle emotion recognition from affective empathy, as participants could share an emotion even if they incorrectly identify it.

Another limitation of the EA task relates to the fact that all clips involved *male* targets talking about autobiographical events – it would have been optimal to have filmed new clips with female targets, as events recalled by female actors may have resonated more with female participants. Furthermore, an own-gender bias in face processing has been found for females, indicating that females are better at recognising female faces than male faces, whereas males recognise male and female faces equally well (see Herlitz and Loven 2013, for a meta-analysis). Despite this, we note that the average scores for emotion recognition were numerically higher in the present female sample than in the previous study using the same task and stimuli in males, even though both study samples viewed male targets. Finally, due to the non-normal distributions of the emotion recognition and affective empathy data, we were unable to use an analytical approach that allowed for sex to be included as a factor of interest in our combined analyses (such as a mixed-design ANOVA). Instead, we had to first compare the four groups and then follow up significant group effects by testing for pairwise group differences.

## Conclusion

In the first study to examine EA in females with CD, which also used more ecologically-valid stimuli than have been used previously, we found that female adolescents with CD displayed reduced affective empathy for happiness and fear relative to TD females. Interestingly, females with the disorder did not exhibit difficulties recognizing others’ emotions, contrasting with the more global deficits observed in males with CD on the same task. On the other hand, our *combined* analyses demonstrated reduced EA in adolescents with CD relative to their TD counterparts, suggesting that males and females with the disorder may find it more difficult to accurately track changes in emotional intensity over time – but such effects are small and relatively large sample sizes are required to detect them.

Although the female CD/CU+ and CD/CU− subgroups did not differ in terms of empathy, and there were no dimensional effects of CU traits within the CD group, higher levels of CU traits were associated with reduced affective empathy within the female TD group. We also found that CU traits were negatively associated with EA when considering the entire female sample (collapsing across the CD and TD groups). While further studies with larger sample sizes and video-clips depicting male and female targets are needed to replicate and extend these results, EA paradigms such as the one used here could be used to assess empathy in clinical settings, as well as evaluating the effectiveness of empathy skills training programmes in youth with CD.

## Electronic supplementary material

ESM 1 (DOCX 20 kb)

ESM 2 (DOCX 15 kb)

ESM 3 (DOCX 13 kb)

ESM 4 (DOCX 19 kb)

ESM 5 (DOCX 15 kb)
