# Supplementary material for: Empathic Accuracy in Female Adolescents with Conduct Disorder and Sex Differences in the Relationship Between Conduct Disorder and Empathy
Source: J Abnorm Child Psychol. 2020 Jun 2;48(9):1155–67. doi: 10.1007/s10802-020-00659-y (PMC7392945; doi:10.1007/s10802-020-00659-y)
Supplement: Supplementary file 1 — (DOCX 20 kb) [file 10802_2020_659_MOESM1_ESM.docx]

Supplementary Table 1. *Demographic characteristics and comorbidity: CD/CU- vs. CD/CU+ comparisons*

|  | CD/CU- (*n* = 13) | CD/CU+  (*n* = 10) | *p* value |
| --- | --- | --- | --- |
|  | *M* (SD) | *M* (SD) |  |
| Age (years) | 16.29 (1.21) | 15.77 (2.09) | .49 |
| Estimated IQ | 90.31 (13.74) | 97.70 (18.66) | .29 |
| Callous-unemotional traits (ICU) | 22.23 (6.25) | 35.50 (4.22) | <.001 |
| Empathy questionnaire (IRI)  Perspective-taking  Fantasy  Empathic concern  Personal distress | 13.67 (4.05)  11.42 (5.66)  17.92 (2.43)  12.25 (4.79) | 10.88 (3.44)  11.50 (5.78)  13.88 (1.55)  10.50 (3.89) | .13  .98  <.001  .40 |
|  | *n* (%) | *n* (%) |  |
| Socioeconomic status ≠  Higher  Lower  Missing | 4 (31)  6 (46)  3 (23) | 4 (40)  5 (50)  1 (10) | .85 |
| Ethnicity  Caucasian  Non-white | 13 (100)  0 (0) | 9 (90)  1 (10) | .24 |
| Psychiatric comorbidity  ADHD  Mood disorder  Anxiety disorder | 1 (8)  3 (23)  3 (23) | 2 (20)  0 (0)  2 (20) | .39  .10  .86 |

*Note:* ≠ Estimated on the basis of parental occupation using National Office of Statistics guidelines; Key: ADHD, attention-deficit/hyperactivity disorder; ICU, Inventory of Callous-Unemotional traits; IQ, intelligence quotient; IRI, Interpersonal Reactivity Index; SD, standard deviation
