# Supplementary material for: Empathic Accuracy in Female Adolescents with Conduct Disorder and Sex Differences in the Relationship Between Conduct Disorder and Empathy
Source: J Abnorm Child Psychol. 2020 Jun 2;48(9):1155–67. doi: 10.1007/s10802-020-00659-y (PMC7392945; doi:10.1007/s10802-020-00659-y)
Supplement: Supplementary file 2 — (DOCX 15 kb) [file 10802_2020_659_MOESM2_ESM.docx]

**Supplementary Table 2.** *Empathic accuracy data descriptive statistics: Female CD/CU- vs. CD/CU+ group comparisons*

| Emotion | CD/CU- (*n* = 13)  Mean correlation (*r*) (SE) | CD/CU+ (*n* = 9^a^)  Mean correlation (*r*) (SE) |
| --- | --- | --- |
| Sadness  Happiness  Fear  Surprise  Anger  Disgust | .32 (.09)  .42 (.10)  .42 (.12)  .36 (.12)  .21 (.08)  .22 (.14) | .20 (.12)  .19 (.10)  .26 (.12)  .37 (.13)  .15 (.13)  -.06 (.15) |

*Note:* Mean scores were transformed back to correlation coefficient scores (*r*) from Fisher’s Z for ease of interpretation. ^a^ Data were unavailable for one CD/CU+ subject due to technical difficulties. Key: CD/CU-, Conduct Disorder with low levels of callous-unemotional traits; CD/CU+, Conduct Disorder with higher levels of callous-unemotional traits; SE, standard error.
