# Supplementary material for: Empathic Accuracy in Female Adolescents with Conduct Disorder and Sex Differences in the Relationship Between Conduct Disorder and Empathy
Source: J Abnorm Child Psychol. 2020 Jun 2;48(9):1155–67. doi: 10.1007/s10802-020-00659-y (PMC7392945; doi:10.1007/s10802-020-00659-y)
Supplement: Supplementary file 3 — (DOCX 13 kb) [file 10802_2020_659_MOESM3_ESM.docx]

| **Supplementary Table 3.**  *Demographic characteristics and comorbidity: group comparisons for the analyses including males* | | | | | | |
| --- | --- | --- | --- | --- | --- | --- |
|  | TD Males^1^ (*n* = 29) | CD Males^2^  (*n* = 23) | TD Females^3^ (*n* = 29) | CD Females^4^  (*n* = 23) |  |  |
|  | *M* (SD) | | | | *F* | Post-hocs |
| Age (years) | 16.33 (1.42) | 16.15 (1.72) | 16.22 (1.94) | 16.06 (1.63) | .12 | - |
| IQ | 101.34 (10.01) | 90.22 (10.85) | 100.17 (12.66) | 93.52 (16.11) | 4.65** | 1, 3 > 2; 1 > 4 |
| Callous-unemotional traits (ICU) | 24.59 (7.26) | 30.09 (8.87) | 19.19 (6.51) | 28.00 (8.59) | 9.46*** | 1, 2, 4 > 3 ; 2 > 1 |
| Empathy (IRI) |  |  |  |  |  |  |
| Perspective taking | 15.76 (3.76) | 13.35 (5.58) | 15.79 (5.04) | 12.55 (3.98) | 3.10* | 1, 3 > 4 |
| Fantasy | 14.21 (5.69) | 11.52 (5.99) | 14.72 (5.07) | 11.45 (5.56) | 2.40 | - |
| Empathic concern | 17.76 (4.15) | 13.48 (5.53) | 17.48 (3.61) | 16.30 (2.90) | 5.47** | 1, 3, 4 > 2 |
| Personal distress | 11.24 (3.53) | 11.57 (6.01) | 11.24 (2.92) | 11.55 (4.43) | .46 | - |
| Total IRI | 58.97 (11.82) | 49.91 (13.68) | 65.10 (8.69) | 56.60 (12.28) | 7.53*** | 1, 3 > 2; 3 > 1, 4 |
|  | *n* (%) | | | | *χ²* |  |
| Socioeconomic status ≠ |  |  |  |  |  |  |
| Higher | 18 (62) | 6 (26) | 15 (52) | 8 (35) | 8.74 | - |
| Lower | 7 (24) | 13 (57) | 10 (34) | 11 (48) |  |  |
| Missing | 4 (14) | 4 (17) | 4 (14) | 4 (17) |  |  |
| Psychiatric comorbidity |  |  |  |  |  |  |
| ADHD | 0 (0) | 8 (35) | 0 (0) | 3 (13) | 2.99 | - |
| Mood disorder | 0 (0) | 3 (13) | 1 (3) | 3 (13) | 1.94 | - |
| Anxiety  disorder | 0 (0) | 1 (4) | 1 (3) | 5 (22) | 6.05* | 3 < 4 |
| Substance  abuse | 0 (0) | 2 (9) | 0 (0) | 0 (0) | - | - |
| Alcohol abuse | 0 (0) | 1 (4) | 0 (0) | 0 (0) | - | - |
|  |  |  |  |  |  |  |

*Note:* ≠ Estimated on the basis of parental occupation using National Office of Statistics guidelines; **p* < .05; ***p* < .01; ****p* < .001. Key: ADHD, attention-deficit/hyperactivity disorder; CD, Conduct Disorder; ICU, Inventory of Callous-Unemotional traits; IQ, intelligence quotient; IRI, Interpersonal Reactivity Index; TD, typically-developing
