# Supplementary material for: Empathic Accuracy in Female Adolescents with Conduct Disorder and Sex Differences in the Relationship Between Conduct Disorder and Empathy
Source: J Abnorm Child Psychol. 2020 Jun 2;48(9):1155–67. doi: 10.1007/s10802-020-00659-y (PMC7392945; doi:10.1007/s10802-020-00659-y)
Supplement: Supplementary file 4 — (DOCX 19 kb) [file 10802_2020_659_MOESM4_ESM.docx]

|  | TD Males (*n* = 29) | CD Males (*n* = 23) | TD Females (*n* = 28^a^) | CD Females (*n* = 22^a^) |
| --- | --- | --- | --- | --- |
| Emotion | Mean correlation (*r*) (SE) | | | |
| Sadness | .47 (.04) | .42 (.05) | .36 (.04) | .27 (.07) |
| Happiness | .47 (.05) | .51 (.07) | .42 (.06) | .33 (.07) |
| Fear | .51 (.04) | .42 (.06) | .34 (.07) | .35 (.08) |
| Surprise | .45 (.05) | .27 (.06) | .38 (.06) | .36 (.08) |
| Anger | .31 (.05) | .27 (.05) | .29 (.05) | .18 (.07) |
| Disgust | .46 (.07) | .32 (.09) | .33 (.09) | .10 (.11) |

**Supplementary Table 4.** *Empathic accuracy descriptive statistics: group comparisons for the analyses including males*

*Note:* Mean scores were transformed back to correlation coefficient scores (*r*) from Fisher’s Z for ease of interpretation. Scores could range from -1 to 1 with higher scores representing higher levels of empathic accuracy. Key: CD, Conduct Disorder; SE, standard error; TD, typically-developing. ^a^ Empathic accuracy data were unavailable for one TD and one CD female due to technical difficulties.
